# Corrigendum to “A facile room temperature method to recycle Cd from CdS” [Heliyon 9(4) (April 2023) e15229]

**DOI:** 10.1016/j.heliyon.2023.e15877

**Published:** 2023-05-06

**Authors:** Huazhang Feng, Tingting Xu, Yuanmin Zhu, Yanping Chen, Jingyun Su, Enna Ha, Ran Jia, Kan Zhang, Lei Ma, Luyang Wang

**Affiliations:** a College of New Materials and New Energies, Shenzhen Technology University, Shenzhen, Guangdong, 518118, PR China; b MIIT Key Laboratory of Advanced Display Material and Devices, College of Materials Science and Engineering, Nanjing University of Science and Technology, Nanjing, 210094, China; c Institute of Theoretical Chemistry, Jilin University, Changchun, 130023, China; d College of Health Science and Environmental Engineering, Shenzhen Technology University, Shenzhen, 518118, China; e School of Material Science and Engineering, Dongguan University of Technology, Dongguan, 523106, China

In the original published version of this article, the acknowledgements were incorrectly listed as ‘The National Natural Science Foundation of China (NSFC) is an institution directly under the jurisdiction of the State Council, tasked with the administration of the National Natural Science Fund from the Central Government’. The correct acknowledgements is ‘This work is supported by the 10.13039/501100001809National Natural Science Foundation of China (Nos. 12004156 and 21902104), Natural Science Foundation of Top Talent of SZTU (Nos. 2019010801003 and 2019010801006), the Science and Technology Research Program of Education Department of Jilin Province (No. JJKH20200939KJ), Shenzhen Basic Research Fund (No. JCYJ20190809181601639 and 20220717183323001) and Foundation for Young Innovative Talents in Higher Education of Guangdong (Nos. 2020KQNCX069 and 2018KQNCX401)’. The authors apologize for the errors. Both the HTML and PDF versions of the article have been updated to correct the errors.

## Declaration of competing interest

The authors declare no conflict of interest.

